# Harnessing Apoptotic Cell Clearance to Treat Autoimmune Arthritis

**DOI:** 10.3389/fimmu.2017.01191

**Published:** 2017-10-09

**Authors:** Philippe Saas, Francis Bonnefoy, Eric Toussirot, Sylvain Perruche

**Affiliations:** ^1^INSERM, EFS BFC, UMR1098, Interactions Hôte-Greffon-Tumeur/Ingénierie Cellulaire et Génique, Fédération Hospitalo-Universitaire INCREASE, LabEx LipSTIC, Université Bourgogne Franche-Comté, Besançon, France; ^2^INSERM CIC-1431, University Hospital of Besançon, Clinical Investigation Center in Biotherapy, Fédération Hospitalo-Universitaire INCREASE, LabEx LipSTIC, Besançon, France; ^3^Department of Rheumatology, University Hospital of Besançon, Besançon, France; ^4^Department of Therapeutics, Université Bourgogne Franche-Comté, UPRES EA 4266, Pathogenic Agents and Inflammation, Besancon, France

**Keywords:** apoptotic cells, rheumatoid arthritis, collagen-induced arthritis, macrophages, regulatory T cells, efferocytosis, cell-based therapy, biologic DMARD

## Abstract

Early-stage apoptotic cells possess immunomodulatory properties. Proper apoptotic cell clearance during homeostasis has been shown to limit subsequent immune responses. Based on these observations, early-stage apoptotic cell infusion has been used to prevent unwanted inflammatory responses in different experimental models of autoimmune diseases or transplantation. Moreover, this approach has been shown to be feasible without any toxicity in patients undergoing allogeneic hematopoietic cell transplantation to prevent graft-*versus*-host disease. However, whether early-stage apoptotic cell infusion can be used to treat ongoing inflammatory disorders has not been reported extensively. Recently, we have provided evidence that early-stage apoptotic cell infusion is able to control, at least transiently, ongoing collagen-induced arthritis. This beneficial therapeutic effect is associated with the modulation of antigen-presenting cell functions mainly of macrophages and plasmacytoid dendritic cells, as well as the induction of collagen-specific regulatory CD4^+^ T cells (Treg). Furthermore, the efficacy of this approach is not altered by the association with two standard treatments of rheumatoid arthritis (RA), methotrexate and tumor necrosis factor (TNF) inhibition. Here, in the light of these observations and recent data of the literature, we discuss the mechanisms of early-stage apoptotic cell infusion and how this therapeutic approach can be transposed to patients with RA.

## Introduction

Apoptotic cells, at least at their early stage, possess immunomodulatory properties [please refer to recent reviews (1, 2)]. These cells are generated by a process called apoptosis (primarily termed programmed cell death), initially defined on morphological features (3). Today, apoptotic cells can be characterized at different levels by biochemical and genetic methods (4). Different forms of cell death have been identified so far (4). Early-stage apoptotic cells, as defined in this review, correspond to cells characterized *in vitro* (*i.e*., before *in vivo* administration) that express phosphatidylserine (PtdSer) at their cell surface and keep the ability to exclude vital dyes [propidium iodide (PI) or 7-aminoactinomycin D (7-AAD)]. This exclusion means that these early-stage apoptotic cells conserve their cell membrane integrity. Exposure of PtdSer on the surface of early-stage apoptotic cells allows their rapid removal by macrophages (5), thus preventing apoptotic cell “explosion” and the release of pro-inflammatory factors. At steady state, efficient apoptotic cell clearance by macrophages (a process called efferocytosis) has been shown to limit subsequent immune responses. Initially, this clearance of apoptotic cells by macrophages has been identified using apoptotic thymocytes (6). This observation has been then extended by Savill and colleagues to the removal of apoptotic neutrophils (7). This seminal work serves as a basis to explain later on, the resolution of inflammation (8). These interactions of apoptotic cells with monocytes or macrophages are associated with a decreased capacity to produce pro-inflammatory cytokines together with the ability to produce anti-inflammatory factors. This has been reported at the end of the nineties (9), and this process is now called macrophage reprogramming. For timelines of the history of apoptosis in inflammation, readers can refer to two recent reviews (10, 11). In contrast, altered efferocytosis has been associated with autoimmune diseases. For instance, a deficiency in the last step of efferocytosis, namely the digestion of apoptotic cell materials by macrophages (*i.e*., a defect in intracellular DNase II), has been shown to be responsible for a polyarthritis syndrome similar to rheumatoid arthritis (RA) (12). Based on their immunomodulatory properties, early-stage apoptotic cells have been used to prevent unwanted inflammatory responses in different experimental models of autoimmune diseases or transplantation [for a recent review, please refer to Ref. (2)]. Hence, prevention of arthritis by early-stage apoptotic cell injection has been reported in different mouse and rat models (13–15). Moreover, this approach of apoptotic cell infusion has been shown to be feasible without any toxicity in patients undergoing allogeneic hematopoietic cell transplantation (16). However, whether early-stage apoptotic cell infusion can be used to treat ongoing inflammatory disorders has not been reported extensively. Xenogeneic human apoptotic cell administration 3 days after sepsis initiation in mouse models stimulates the resolution of acute inflammation (17). This therapeutic effect of apoptotic cell infusion in sepsis has been confirmed in lipopolysaccharide-induced endotoxic shock as well as in cecal ligation and puncture sepsis (18). Furthermore, donor apoptotic cell infusion can interfere with acute graft rejection in a mouse model of allogeneic cardiac transplantation (19). Recently, we have provided evidence that apoptotic cell infusion is able to control, at least transiently, ongoing collagen-induced arthritis (CIA) (20). Interestingly, the efficacy of this approach is not altered by the association with two standard treatments of RA, methotrexate (MTX) and tumor necrosis factor (TNF) inhibition (20). Here, in the light of these observations and recent data of the literature, we discuss the mechanisms of this therapeutic approach and how it can be transposed to patients with RA.

## Current Knowledge in RA Pathophysiology and Therapeutic Approaches

Rheumatoid arthritis is an autoimmune disorder characterized by a chronic inflammation of the synovial joints leading to the destruction of cartilage, bone, and ligaments (21). However, RA is a heterogeneous syndrome as attested by genetic studies (22, 23). The pathophysiology of RA implicates several immune cell subsets belonging to both innate (e.g., neutrophils, macrophages) and adaptive immunity (*i.e*., T and B cells). At the inflammatory site, the synovial lining becomes thickened due to an infiltration of macrophages and the proliferation of resident synovial fibroblasts (also called fibroblast-like synoviocytes). At the end of the eighties, massive infiltration of neutrophils and macrophages was reported in the joints of patients suffering from acute sterile arthritis, among whom RA patients (7). These data serve as a basis for our current understanding of the resolution step of inflammation and identify neutrophils and macrophages as key players in RA pathogenesis. While the exact etiology of RA is still unknown, macrophage activation leading to local inflammatory cytokine secretion in the joints can be considered as one of these etiologies (12). Therapeutic approaches triggering these inflammatory cytokines (*i.e*., TNF-α, IL-1β, or IL-6) have been used to treat RA patients (24, 25). Despite recent significant advances in the characterization of monocyte and macrophage subsets, the origin of macrophages infiltrating or present in the joint remains to be explored in RA (26). Indeed, the origin of joint macrophages (tissue-resident *versus* derived from blood Ly6C^high^ monocytes) depends on the considered arthritis models (26). Recently, it has been shown that neutrophils may participate in RA pathophysiology through the formation of neutrophil extracellular traps (NET), which consist of DNA fibers associated with a large amount of antimicrobial peptides (e.g., LL37) and nuclear proteins (e.g., high mobility group box-1). This has been reported in RA, as well as in experimental models such as CIA (27–29). Formation of NET by neutrophils during arthritis provides a pro-inflammatory loop *via* the secretion of pro-inflammatory cytokines (28). Dendritic cells (DC)—both conventional DC (cDC) and plasmacytoid DC (pDC)—may also play a role in RA pathophysiology. For instance, pDC are present in the synovial fluid of RA patients (30–32). Pro-inflammatory pDC aggravates ongoing CIA (33). Activation of cDC by NET may be also involved in arthritis pathogenesis (29). Pathogenic CD4^+^ helper T (Th) and cytotoxic CD8^+^ T cells have been also implicated in RA, while the exact target of these cells has not been fully characterized. However, autoreactive CD4^+^ T cells specific to citrullinated epitopes with a memory and/or effector phenotype have been identified in some RA patients (34). Concerning CD8^+^ T cells, Epstein–Barr virus (EBV)-derived antigens can be targeted antigens in RA since high expression of EBV markers is present in RA synovium (35). These cytotoxic T cells can mediate joint damage, but in all cases, inflammatory CD4^+^ Th cells are required. Both interferon-γ (IFN-γ)-secreting Th1 and IL-17-producing Th17 cells (36) are involved in RA pathogenesis. They are driven mainly by macrophage cytokines consisting of TNF and IL-12 *versus* IL-23 for Th1 and Th17 cell polarization, respectively (26). These two Th cell polarization pathways occur in the absence of adequate immune regulation, since an altered regulatory CD4^+^ T cell (Treg) response is another feature of RA (37). Finally, concerning B cell responses, a high frequency of circulating polyspecific B cell clones has been found in RA patients (23). However, it is unclear how such B cells contribute to RA disease. The reversion of anergic autoreactive B cells under inflammatory conditions has been suggested to participate in RA pathogenesis (23). Nevertheless, the implication of auto-antibodies in RA pathophysiology is highlighted by the two major biological tests performed for RA diagnosis: rheumatoid factor (RF) and anti-citrullinated protein antibody (ACPA) detection (35). RF is involved in the formation of immune complex (IC) that induces complement activation responsible for its consumption and generates non-resolving inflammation observed in RA (35, 38). Non-resolving inflammation significantly contributes to RA pathogenesis (38). Citrullinated proteins result from arginine-containing proteins modified by deimination mediated by intracellular enzymes, called peptidyl-arginine deiminases. NET produced by neutrophils can be an additional source of citrullinated autoantigens (28, 39). These resultant citrullinated proteins could be the antigenic component of IC driving RF production (35) and become the targets of autoantibody responses (35), as well as autoreactive CD4^+^ T cells (34). Furthermore, ACPA are T cell-dependent immunoglobulin G auto-antibodies, and thus, follicular helper T cells may help B cell activation in ACPA-positive RA (34). Thus, several immune mechanisms and immune cell subsets participate in RA pathophysiology and represent targets for therapeutic strategies, such as apoptotic cell infusion.

Today, no causal treatment of RA is available, since RA is still a chronic inflammatory disorder of unknown cause. Hence, there is currently no curative treatment for RA and treatment has to be initiated for prolonged periods of time if not for life (40). The European League Against Rheumatism organization recommends that patient starts treatment with conventional synthetic disease-modifying anti-rheumatic drugs (csDMARD) in combination with corticosteroids, followed by biologic DMARD (bDMARD) in the case of a non-response to the initial regimen and the presence of poor prognosis markers (41). Treatment of RA aims to limit disease symptoms, delay or prevent future joint destruction, and target low disease activity (LDA) or remission. According to a recent review (40), LDA is a state in which the progression of joint damage is minimal and physical function, quality of life and work capacity are preserved. Low-dose MTX is the traditional csDMARD administered weekly either alone, or in combination with corticosteroid or bDMARD. While the precise molecular mechanism of MTX remains to be determined, MTX alone has been proven safe and efficient in RA (42). However, nearly a quarter of patients treated with MTX have to discontinue their treatment because of inadequate responses, adverse effects (e.g., hepatic, gastrointestinal, hematological, renal, or pulmonary toxicity), or both (43, 44). Biologic agents targeting inflammatory cytokines, such as anti-TNF therapy, combined with MTX have significantly improved the treatment of RA (24, 40). However, again, some RA patients are refractory or have contraindications to receive these agents (44, 45). The proportion of patients who do not respond favorably to TNF inhibitors is estimated between 30 and 40% (24). Only few RA patients achieve complete remission after such treatment (24). Moreover, adherence to treatment with biologic agents is moderate with only around 60% of RA patients respecting treatment regimens over a 1- or 2-year period (46). This requires frequently a switch to another form of treatment (40, 46). Biologic agents targeting different modes of action have been developed, and are now available in RA. This consists in TNF blocking agents, IL-6 or IL-1 inhibitors, T-cell costimulatory modulators, or B-cell depletion therapies (25). However, despite this multitude of treatments, treatment failure occurs and RA patients are still in need of new treatment modalities (25). Finally, it should be mentioned that combinations of bDMARD acting on different therapeutic targets (*i.e*., TNF, IL-6, or B cells) usually do not increase efficacy, but are more toxic (47). Overall, new therapeutic strategies are needed in RA among which cell-based therapies could be proposed, such as apoptotic cell infusion.

## The Disease-Modifying Anti-Rheumatic Potential of Apoptotic Cell Infusion

In this section, we will describe the mechanisms by which early-stage apoptotic cell infusion may treat ongoing arthritis. Based on our recent data (20), we will focus on the resolution of inflammation, antigen-presenting cells (APC), including DC subsets and macrophages, as well as CD4^+^ T cell polarization. Data obtained using apoptotic cell infusion as prevention of arthritis (13–15, 48) will be also considered to shed light on these mechanisms.

### Lessons from Preclinical Arthritis Models

**Figure 1:**
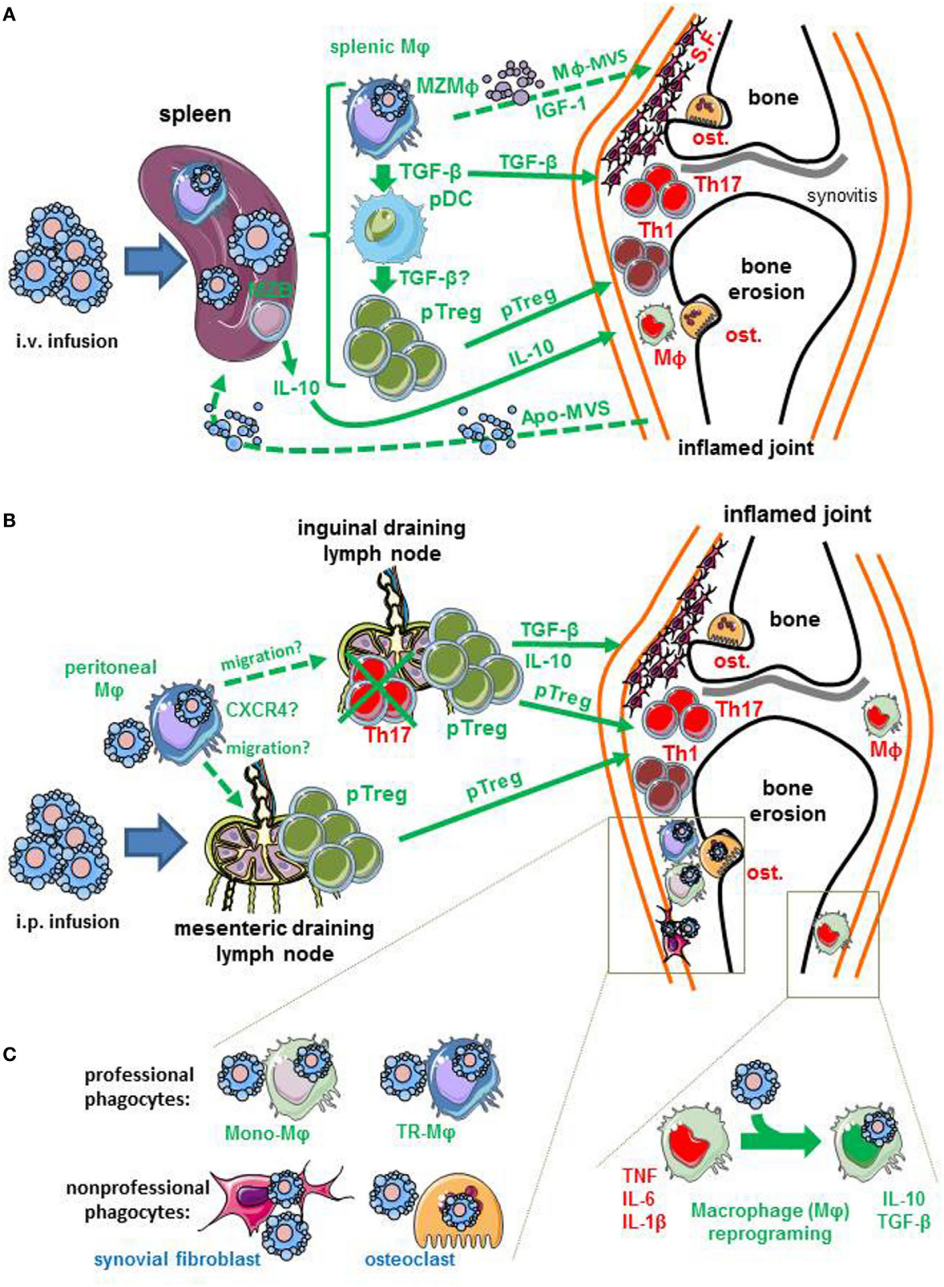
Potential immune mechanisms induced by early-stage apoptotic cell infusion in arthritis. Apoptotic cells are infused by two routes: the intravenous (i.v.) and the intraperitoneal (i.p.) routes. **(A)** Apoptotic cells infused intravenously are certainly eliminated by the spleen, and more specifically marginal zone (MZ) macrophages (MZMφ). Splenic Mφ plays a critical role in the effect of i.v. apoptotic cell infusion. These cells may act on inflamed joint by soluble factors (e.g., IL-10 or TGF-β), the generation of peripheral regulatory CD4^+^ T cells (pTreg) that migrate to the inflamed joints. Alternatively, the immunosuppressive mechanisms identified in the spleen (pro-Treg pDC, anti-inflammatory Mφ or pTreg) can reflect the transfer of tolerance generated in the joints by apoptotic cells to the spleen. Apoptotic materials, such as apoptotic-derived microvesicles (Apo-MVS) have been proposed to mediate this transfer of tolerance from peripheral tissues to the spleen. Finally, splenic Mφ may imprint local joint phagocytes *via* the release of insulin-like growth factor (IGF)-1 and macrophage-derived microvesicles (Mφ-MVS). **(B)** Apoptotic cells injected intraperitoneally can be eliminated by peritoneal Mφ. These cells may migrate to lymph nodes, including mesenteric lymph nodes, and maybe, inguinal draining lymph nodes to stimulate the generation of pTreg. This migration may be guided by the CXCR4/CXCL12 axis. Peripheral Treg generated in the draining lymph nodes are able to reach inflamed joints. **(C)** Infused apoptotic cells may reach the inflamed joints, and be eliminated by local joint Mφ. These Mφ can be either tissue-resident Mφ (TR-Mφ) that have colonized the joints during embryogenesis or blood monocyte-derived Mφ (Mono-Mφ) that have migrated in response to inflammatory signals. The uptake of apoptotic cells by these joint Mφ may be responsible for Mφ reprogramming, that corresponds to the capacity to produce anti-inflammatory factors (e.g., IL-10, TGF-β, or pro-resolving lipid mediators) and lose their ability to secrete pro-inflammatory cytokines [*i.e*., tumor necrosis factor (TNF), IL-1β or IL-6]. Non-professional phagocytes, such as osteoclasts (ost.) or synovial fibroblasts (S.F.) may also remove apoptotic cells. Deleterious effectors (TNF, Mφ, osteoclasts, synovial fibroblasts, Th1, or Th17 cells) of arthritis present in the inflamed joints are written in red font, while factors or effectors triggered by apoptotic cell infusion are written in green font. Dotted arrows correspond to hypotheses, whereas solid arrows represent data obtained in experimental arthritis models. For references, see the text.

Early-stage apoptotic cells have been injected in arthritis experimental models before the disease is fully established or at time of immunization with the autoantigen (13–15, 48). This is not relevant to the clinical situation, and contrasts with our recent study in which early-stage apoptotic cells are infused when CIA reaches a clinical score of 8 out of 16 (20). Therefore, one may distinguish the prophylactic *versus* the therapeutic effect of apoptotic cell infusion (Table 1). To date, one limitation is that only one experimental model has been tested for the therapeutic effect (20). For the prophylactic effect, several experimental models have been used (13–15). These models recapitulate differently RA pathophysiology. An absence of prevention has been reported in the serum transfer-induced arthritis (STIA) (13) in which arthritis is induced by the intraperitoneal (i.p.) injection of K/BxN serum in C57BL/6 mice (49) (Table 1). This STIA model recapitulates the effector phase of human RA, but is independent of the adaptive immune response (49, 50). Thus, this suggests that the prophylactic effect of apoptotic cell infusion modulates rather the adaptive immune response, such as CD4^+^ T cell polarization. The model consisting in injecting streptococcal cell wall (SCW) in Lewis rats is induced by a single i.p. injection of SCW fragments (51). This results in a first T cell-independent phase followed by a chronic inflammatory phase that is T cell-dependent and associated with the production of high levels of inflammatory cytokines (51). This results in erosive cartilage damage in the joints (51). In the prophylactic approach using early-stage apoptotic cell infusion, both phases were significantly reduced but the effect was more impressive or pronounced on the chronic phase (14). This is consistent with an impact of apoptotic cell infusion on inflammatory cytokine secretion by macrophages affecting the first phase and on Treg increase modulating the second chronic phase (14) (Table 1). Methylated bovine serum albumin (mBSA)-induced arthritis in C57BL/6 mice belongs to antigen-induced arthritis. In this model, arthritis results from IC-mediated inflammation followed by articular T cell-mediated responses. However, this model does not recapitulate the endogenous breach of tolerance that is typical of RA pathogenesis. This represents a limitation in applicability to RA (50). The prophylactic effect of apoptotic cell infusion has been observed in this model (15, 48) (Table 1). This effect is dependent on natural IgM and IL-10 secretion (15). Finally, the CIA model in DBA/1 mice has been used to evaluate the prophylactic and therapeutic effect of apoptotic cell infusion (13, 20) (Table 1). This mouse model shares with human RA several clinical (*i.e*., erythema and edema), histopathological (*i.e*., synovitis, pannus formation, cartilage, and bone erosion), as well as immunological features (51). These features consist in the breach of tolerance with the implication of pathogenic T cells associated with the production of inflammatory cytokines (e.g., TNF), as well as the production of auto-antibodies against self-antigens and collagen (50). Some drawbacks have been evoked for this CIA model. The main drawback is that CIA constitutes only an acute model in contrast to the SCW model (52). Nevertheless, all these models are relevant to some features of RA (49–51), and most of them have been used to test drugs now in clinical development for RA (51). Now, we will highlight some immune mechanisms (Figures 1A–C) and propose future investigations.

**Table 1:** Effects (therapeutic *versus* prophylactic) of early-stage apoptotic cell infusion in arthritis models.

Experimental models	Effects on disease	Administration route	Characteristics of infused apoptotic cells	Immune mechanisms	Reference
CIA (DBA/1)	Therapeutic	i.v.	Syngeneic thymocytes, 5 or 15 × 10^6^, early-stage apoptotic cells (70–85% AxV^+^/7-AAD^−^ and <10% 7-AAD^+^)	Pro-Treg splenic macrophages; splenic cDC and pDC resistant to TLR ligand stimulation—pro-Treg splenic pDC; induction of auto-Ag-specific Treg in the DLN; reduction of pathogenic anti-collagen auto-Abs; depend on TGF-β	Bonnefoy *et al*. (20)

CIA (DBA/1)	Prophylactic	i.v. or i.p.	Syngeneic thymocytes, 2 × 10^7^ (total 3 consecutive days), early-stage apoptotic cells (mean: 43% of AxV^+^ and <5% PI^+^)	IL-10-producing splenic and PLN CD4^+^ T cells; reduction of IFN-γ secreting CD4^+^ T cells; IL-10-producing MZB cells; reduction of pathogenic anti-collagen auto-Abs	Gray *et al*. (13)

STIA (C57BL/6)	No effect	i.v. or i.p.	Same as above	No prophylactic effect but T cell-independent model (50)	Gray *et al*. (13)

SCW (Lewis rats)	Prophylactic	i.p.	Mouse thymocytes, 2 × 10^8^, early-stage apoptotic cells (90–95% AxV^+^/7-AAD^−^)	Decrease of peritoneal macrophage pro-inflammatory response (tumor necrosis factor); increase of blood and DLN Treg; depend on TGF-β	Perruche *et al*. (14)

mBSA (C57BL/6)	Prophylactic	i.v.	Syngeneic thymocytes, 3 × 10^7^, 3 consecutive days, early-stage apoptotic cells (60–80% AxV^+^/PI^−^)	Decrease of DLN Th17, but not Th1 cells; increase of DLN IL-10-producing T cells; IL-10-producing MZB cells; depend on natural IgM	Notley *et al*., 2011 (15)

mBSA (C57BL/6)	Prophylactic	i.v.	Syngeneic DC, 2 × 10^7^, 3 consecutive days, early-stage apoptotic cells (60–75% AxV^+^/PI^−^ and 8–11% PI^+^)	Activated apoptotic cells induce IL-6 and prevent TGF-β-mediated prevention of arthritis	Notley *et al*., 2015 (48)

*7-AAD, 7-aminoactinomycin D; Ab, antibody; Auto-Ag, autoantigen; AxV, annexin-V; cDC, conventional DC; CIA, collagen-induced arthritis; DC, dendritic cell; DLN, inguinal draining lymph node; i.p., intraperitoneal; i.v., intravenous; mBSA, methylated bovine serum albumin; MZB, marginal B cells; pDC, plasmacytoid DC; PI, propidium iodide; PLN, peripheral lymph node; SCW, streptococcal cell wall; STIA, serum transfer-induced arthritis (i.e., intraperitoneal injection of K/BxN serum in C57BL/6 mice) (49); TLR, toll like receptor; Treg, regulatory CD4^+^ T cells*.

### Effects on Macrophages and Resolution of Inflammation

One of our hypotheses concerning the use of early-stage apoptotic cell infusion to treat ongoing arthritis was that reintroducing apoptotic cells in a context of non-resolving inflammation—a key feature in RA (38)—may force macrophage reprogramming after apoptotic cell uptake and stimulate the resolution of inflammation with a decrease of pro-inflammatory cytokines (Figure 1). Macrophage reprogramming following efferocytosis stimulates anti-inflammatory factors (e.g., TGF-β or IL-10) and reduces the pro-inflammatory ones (e.g., TNF or IL-1β) (9). The importance of anti-inflammatory cytokines, such as TGF-β (14, 20, 48) and IL-10 (13, 15), was shown in the prevention or treatment of experimental arthritis (Table 1). In addition, reduction of TNF after apoptotic cell infusion in the SCW model has been also reported (14) (Table 1). In the therapeutic approach using intravenous (i.v.) apoptotic cell infusion, macrophages sorted from the spleen [*i.e*., the main site where blood-borne apoptotic cells are eliminated (53, 54)] induce the polarization of naive CD4^+^ T cells toward a Treg phenotype. Altogether, this sustains our initial hypothesis.

Now, we want to discuss the implications of macrophages in the beneficial effects of apoptotic cell infusion in the light of recent data from the literature. The critical macrophage subset for this beneficial effect can be: (*i*) splenic macrophages, as identified after i.v. apoptotic cell infusion (20), (*ii*) peritoneal macrophages, as shown after i.p. apoptotic cell infusion (14), or perhaps (*iii*) macrophages present in the joint (Figure 1). This may concern tissue-resident macrophages or monocyte-derived macrophages (26) (Figure 1).

#### Splenic Macrophages

Professional circulating phagocytes, and in particular monocyte-derived macrophages, are guided by “find-me” signals released by dying cells in order to remove apoptotic cells (55). But here, in the case of apoptotic cell infusion to prevent or treat arthritis, injections have been performed, not in the joint, but at distant sites, either i.v. (13, 15, 20) or i.p. (13, 14) (Figure 1). The spleen is the main blood filter (53, 54), and marginal zone macrophages of the spleen are specialized in the uptake of blood-borne apoptotic leukocytes (56). Thus, how can splenic macrophages act on the inflamed joint? First, it can occur by the release of anti-inflammatory cytokines that exert a systemic effect affecting inflamed joints (Figure 1A). Alternatively, immune cells generated in the spleen (e.g., Treg) may migrate to the inflamed joints and limit/control inflammation (Figure 1A). Second, the spleen is a site of immune tolerance induction and can be alerted—or affected—*via* apoptotic “remnants” (including apoptotic cells, or apoptotic materials, such as apoptotic bodies or microvesicles) (54) released by distant tissues during normal cell turn-over (54). This transfer of tolerance from peripheral tissues to the spleen exists also under chronic inflammatory conditions (54). When the functions of splenic macrophages were assessed *ex vivo* after i.v. infusion of apoptotic cells in the setting of arthritis models (20), it is possible that we measured the consequence (*i.e*., the transfer of tolerance from the joint to the spleen) and not the cause of clinical improvement. Third, based on data obtained in the lungs (57, 58), splenic macrophages phagocyting infused apoptotic cells may release insulin-like growth factor-1 (IGF-1) and macrophage-derived microvesicle (Mφ-MVS) (57) targeting joint-infiltrating immune cells. Extracellular vesicles emitted by macrophages have been shown to export an anti-inflammatory signal to distant cells (58). It remains to be determined which one of these three hypotheses (Figure 1A) is responsible for the beneficial effect in arthritis models.

#### Peritoneal Macrophages

Peritoneal macrophages are affected by i.p. infusion of early-stage apoptotic cells in the SCW (14) or the CIA (13) model (Table 1). Recently, it has been reported that macrophages phagocyting apoptotic cells acquire CXCR4 expression and the capacity to migrate in response to CXCL12 (59). This may explain the migration of the so-called “satiated” macrophages to draining lymph nodes after efferocytosis (60). This corresponds to an additional mechanism to export the anti-inflammatory response from tissues where cells die to draining lymph nodes and to participate to the maintenance of tolerance. It remains to be determined whether peritoneal macrophages migrate to draining lymph nodes in the setting of arthritis treatment by apoptotic cell infusion, and if they are then responsible for the modulation of T cell subsets in these lymph nodes. In support of this hypothesis, several modifications of T cell subsets in inguinal draining lymph nodes have been reported in arthritis models (13–15, 20) (Table 1; Figure 1B).

#### Joint Macrophages

In steady state, tissue-resident macrophages are the predominant phagocyting cells in the different tissues analyzed (*i.e*., the bone marrow, spleen, intestine, liver, and the interstitial space of the lungs) (61). This may be related to the expression of an enzyme called, 12/15-Lipoxygenase (12/15-LOX), expressed by tissue-resident macrophages that confines apoptotic cell removal by these resident macrophages and blocks apoptotic cell uptake by inflammatory Ly6C^high^ monocyte-derived macrophages (62). This mechanism may be responsible for the non-immunogenic removal of apoptotic cell-derived antigens in steady state (62). The anti-inflammatory phenotype imprinted by apoptotic cell phagocytosis in resident macrophages is only partially preserved across the different tissues analyzed (61). Thus, based on this elegant study (61), it is not possible to predict the consequences for joint-infiltrating or joint-resident macrophages under inflammatory conditions. The origin of macrophages present in inflamed joint (tissue-resident *versus* derived from monocytes) has not been deciphered to date (26). Whatever the origin of joint macrophages contributing to apoptotic cell clearance in the therapeutic effect of early-stage apoptotic cells (Figure 1C), one may imagine that some mechanisms described for splenic or peritoneal macrophages (Figures 1A,B) may occur. Although the anti-inflammatory response imprinted by apoptotic cell phagocytosis in macrophages in steady state is partially preserved across the different tissues analyzed (61), one may postulate that certain mechanisms may be conserved, such as macrophage reprogramming associated with cytokine secretion. Indeed, the downregulation of *Il1b* transcripts in phagocyting macrophages has been found in all tissues analyzed so far (61). Nevertheless, this remains to be determined specifically in the inflamed joints.

The implication of 12/15-LOX in joint macrophages after apoptotic cell infusion is relevant in arthritis. Indeed, the expression of 12/15-LOX is not always confined to tissue-resident macrophages (63). While this observation is true during steady state, other macrophage subsets, in particular monocyte-derived macrophages, may acquire 12/15-LOX expression in response to cytokines (63) or after interactions with apoptotic cells (60, 63, 64). This is the case of the so-called “alternatively activated” M2 macrophages that express 12/15-LOX in response to the triggering of the IL-4 receptor-α signaling pathway, common to both IL-4 and IL-13 (63). An increase in *Alox15* (*i.e*., the gene encoding 12/15-LOX) mRNA expression in macrophages during the resolution phase of inflammation has been reported in zymosan-induced peritonitis (65). Furthermore, CD11b^low^ “satiated” (*i.e*., apoptotic cell ingesting) macrophages derived from monocytes have been also shown to express high levels of 12/15-LOX and to possibly promote efferocytosis by the production of pro-resolving lipid mediators, such as resolvin D1 (RvD1) (60). Interestingly, the induction of *Alox15* mRNA has been detected in the synovial tissue of inflamed joints of arthritic mice both in STIA (66) and CIA (67) models. The study of the kinetics of *Alox15* mRNA expression in the inflamed limbs is highly interesting, since *Alox15* mRNA transcripts increase during the CIA induction phase, returns to basal levels during the inflammatory phase, and then increase again during the resolution phase (67). This supports an acquisition of 12/15-LOX by macrophages during the resolution phase of inflammation (65), possibly after efferocytosis (60, 64). Moreover, increased *LOX-15* mRNA expression was found in synovial tissues of RA patients (68). The enzyme 12/15-LOX is the murine ortholog of human 15-LOX (63). These enzymes—human 15-LOX and mouse 12–15/LOX—mediate the oxidation of unsaturated fatty acids. Depending on its substrate (e.g., arachidonic, docosahexaenoic, or linoleic acid), 12/15-LOX generates different key lipid products with anti-inflammatory and pro-resolution properties, such as resolvins, protectins, or lipoxins (63, 69). Lipoxin A4 (LXA4) plays a major role in the resolution of inflammation mediated by 12/15-LOX in experimental arthritis (66, 67). Overall, this suggests that 12/15-LOX is expressed in inflamed joints during arthritis and that this enzyme present in joint macrophages may exert an anti-inflammatory role *via* the synthesis of pro-resolving lipid mediators (e.g., RvD1 or LXA4). One can hypothesize that this mechanism may participate in the local therapeutic effect after apoptotic cell infusion.

#### Non-Professional Phagocytes

Apoptotic cells can be eliminated by several subsets of phagocyting cells, including professional, but also non-professional phagocytes (70). The involvement of these phagocytes appears again to be tissue-dependent. For instance, five different professional phagocyte subsets (*i.e*., macrophages and DC subsets) have been recently identified in the intestine (71). Each professional phagocyte subset is dedicated to a specific task (71). Macrophage subsets phagocyting apoptotic intestinal epithelial cells exert an anti-inflammatory response, while CD103^+^ cDC are rather dedicated to drive peripheral Treg (pTreg) in the draining mesenteric lymph nodes (71). However, non-professional phagocytes, mainly epithelial cells, are also important to control apoptotic cell-induced inflammatory responses in the intestine (72) or in the airway (57). In this latter site, non-professional phagocytes (*i.e*., airway epithelial cells) are controlled by factors released by alveolar macrophages, including IGF-1 and Mφ-MVS (57). Thus, an interaction exists between different phagocytes, and thus, macrophages may affect non-professional phagocytes present in the joint when arthritic animals are treated by early-stage apoptotic cell infusion. Among the potential non-professional phagocytes present in the joint (Figure 1C), synovial fibroblasts can be considered as a candidate since fibroblasts are able to uptake apoptotic cells (73) and rabbit synovial fibroblasts have been reported to ingest latex beads in culture (74) or uptake soluble antigen when infused intravenously at high concentrations (75). Osteoclasts are another possibility of non-professional phagocytes for several reasons: (*i*) elevated osteaclast activities have been observed in RA patients (76); (*ii*) dead cells are found engulfed by osteoclasts *in vivo* (77); and (*iii*) osteoclasts are well capable of ingesting apoptotic thymocytes *in vitro* (78) (Figure 1C).

The immune consequences of apoptotic cell removal by non-professional phagocytes depend on the phagocytes considered. Apoptotic cell removal by non-professional phagocytes is usually slower than removal by professional phagocytes, and particularly macrophages. It requires apoptotic cells at a more advanced stage than early-stage apoptotic cells, and may be limited to subcellular fragments rather than the whole dying cell (73). In certain settings, pro-inflammatory chemokines (e.g., MCP-1) are released by these non-professional phagocytes, leading to inflammatory monocyte recruitment (73). In contrast, in other tissues, neighbor non-professional phagocytes participate efficiently in the control of inflammation after apoptotic removal (57, 72). Thus, the role of synovial fibroblasts and/or osteoclasts in the beneficial effect of apoptotic cell infusion has to be studied in the setting of experimental arthritis models.

Genetic manipulation of non-professional phagocytes (*i.e*., epithelial cells) (72) attenuates inflammation *in vivo* at least in the intestine. This was performed in a non T cell-dependent disease, namely dextran sodium sulfate-induced colitis (72). Even if genetic manipulation is not easily transposable from experimental models to patients, this approach (72) does not appear to be appropriate since data obtained in STIA—a T cell-independent disease (50)—show that apoptotic cell infusion is inefficient to prevent this disease.

### Effects on DC

Here, the interactions of apoptotic cells with cDC and pDC in the settings of arthritis will be discussed. The implication of pDC after i.v. apoptotic cell infusion has been initially shown in the bone marrow transplantation (BMT) model (79), and found again in the CIA model with the capacity of *ex vivo* sorted splenic pDC (20) to generate pTreg (Table 1; Figure 1A). Data on the interactions of early-stage apoptotic cells and pDC are certainly easier to interpret than data on cDC. cDC represent different heterogeneous cDC subsets (80), and tools used so far to analyze the impact of apoptotic cell infusion on cDC functions do not allow researchers to separate each subset. For instance, in CD11c/diphtheria toxin (DT) receptor (DTR) transgenic mice, all CD11c^high^ cells are depleted after DT infusion (81). These CD11c^high^ cells consist of cDC (81), but also of other APC subsets having the ability to eliminate apoptotic cells, such as marginal zone and metallophilic macrophages in the spleen (82), sinusoidal macrophages in the lymph node (82), or alveolar macrophages (83). In contrast, pDC have been shown to be spared by depletion after DT administration in CD11c/DTR mice (79). It is known that depending on the considered cDC subsets, the response against apoptotic cells can be the opposite, with splenic lymphoid-resident cDC implicated in tolerance induction (73) while a particular subset of cDC localized at barrier surfaces (e.g., the intestine, the lungs, and the skin) boosts inflammatory responses *via* a PtdSer receptor CD300a (84). Thus, the role of cDC in the therapeutic effect of apoptotic cell infusion in the setting of arthritis has to be further explored. Nevertheless, in the therapeutic approach using the CIA model, we observed that the addition of anti-TNF therapy to apoptotic cell infusion is able to generate *ex vivo* sorted splenic CD11c^+^ cDC stimulating the polarization of Treg (20). The activation of draining lymph node cDC by NET exacerbated Th1-, but not Th17-, mediated autoimmune responses in CIA (29). This was confirmed *in vitro* by the maturation of human monocyte-derived DC or mouse bone marrow-derived DC in response to NET isolated from CIA mice or RA patients, respectively (29). Interestingly, it was reported that apoptotic cell clearance by neutrophils reduced NET formation (85). Apoptotic cell infusion may, therefore, limit cDC activation and subsequent Th1 responses by limiting NET formation.

### Effects on CD4^+^ T Cell Polarization

One of the salient consequences following apoptotic cell clearance is the induction of pTreg. This has been demonstrated after i.v. apoptotic cell infusion (86) or local apoptotic death of epithelial cells (87). The increase of Treg in the spleen following i.v. apoptotic cell infusion has been shown to require TGF-β (79, 86), splenic macrophages, and donor pDC in the setting of BMT (79). TGF-β is also required for Treg polarization after intestinal epithelial cell apoptosis (87). In arthritis models, the induction of pTreg after apoptotic cell infusion is also TGF-β-dependent (14, 20) (Table 1; Figures 1A,B). In the therapeutic CIA model, we took advantage of the presence of an infectious antigen, *Mycobacterium tuberculosis* (MBT), mixed with collagen in the complete Freund’s adjuvant used for the induction of arthritis, to analyze T cell responses against another antigen than the autoantigen (*i.e*., bovine type II collagen). In contrast to the response observed with collagen, a similar cell proliferation against MBT antigen is found between cells from apoptotic cell-treated and untreated CIA mice. Moreover, the suppressive activity of Treg sorted from apoptotic cell-treated arthritis mice is restricted to collagen and not extended to MBT (20). This strongly demonstrated that the infusion of apoptotic cells allows the induction of pTreg *in vivo* with an antigenic specificity restricted to the collagen autoantigen. The same effect (*i.e*., induction of autoantigen-specific pTreg but not infectious antigen-specific Treg) has previously been reported in a similar therapeutic approach based on to the *in vivo* generation of apoptosis (88). Further works are necessary to explain why apoptotic cell infusion favors the induction of autoantigen-specific Treg. Nevertheless, other teams have reported the induction of IL-10-dependent Treg (13, 15) after the prophylactic infusion of apoptotic cells in arthritis models. Thus, this confirms the induction of pTreg after apoptotic cell infusion and may explain the anti-inflammatory effect in the joint whatever the administration route since the generated pTreg may migrate to inflamed joints.

Concerning safety reasons, one has to evoke the high plasticity of CD4^+^ T cells, and in particular, pTreg that have in common with pro-inflammatory Th17 cells the requirement of TGF-β (36, 89). Apoptotic cell-induced pTreg polarization can be influenced by a simultaneous microbial infection providing IL-6 necessary for Th17 differentiation. The coincident production of IL-6 and TGF-β in response to bacteria and apoptotic epithelial cell death, during orogastric bacterial infection, leads to the generation of both bacteria-specific and autoreactive Th17 cells (87, 90). Similarly, the production of IL-6 together with TGF-β has been shown when “activated” apoptotic cells or apoptotic cells containing high amounts of demethylated DNA have been infused in mBSA arthritis model instead of “resting” apoptotic cells rather containing methylated DNA (48, 91). In the same model, the infusion of “resting” apoptotic thymocytes decreases Th17 cells in the inguinal draining lymph nodes (15). This dichotomy between anti-inflammatory pTreg and pro-inflammatory Th17 cells is not so simple, since different Th17 cell subsets have been now described including pro-inflammatory and anti-inflammatory Th17 cells (89). To date, these subsets have not been studied in the settings of apoptotic cell infusion.

### Perspectives and Considerations for Therapeutic Apoptotic Cell Infusion

Here, we will evoke the clinical perspectives of apoptotic cell infusion. This is based on the preclinical data (Table 1), but also on data obtained in the field of cancer research. There is an extensive literature on the immunomodulation by dead and dying cells in the setting of cancer (92). We propose to discuss the critical points to achieve a beneficial therapeutic effect (73). These are the following: (*i*) peripheral blood leukocytes are the easiest and major source of apoptotic cells to consider in human settings, while apoptotic cells used in the preclinical studies were other apoptotic leukocytes [*i.e*., thymocytes (13–15, 20) or DC (48), Table 1] from a practical point of view. In RA patients, cytapheresis has to be considered to achieve a sufficient number of apoptotic cells as it has been done in the clinical trial in the setting of hematopoietic cell transplantation (16). The highest number of apoptotic leukocytes planned to be infused is 210 million of cells per kilogram. This will require to pool two sequential cytaphereses. Donor-derived apoptotic cells (*i.e*., allogeneic cells) will not be considered in the first instance for ethical/regulatory purposes; patient (*i.e*., syngeneic) apoptotic leukocytes are considered as a cell-based product by the French regulatory agency, while apoptotic cells from healthy volunteers correspond to advanced therapy medicinal products. Nevertheless in experimental models, prevention of arthritis is observed independently of the apoptotic cell origin (*i.e*., syngeneic, allogeneic, or even xenogeneic) (2); (*ii*) a tolerogenic signal inducing early-stage apoptotic cells, that is, leukocytes stained by annexin-V but little or no staining with PI or 7-AAD dyes (Table 1). These stimuli correspond to γ- or ultraviolet B (UVB)-irradiation (73, 93). Stimuli inducing apoptotic cell death have been particularly studied in the field of cancer research in order to generate immunogenic dying tumor cells to favor tumor rejection. A recent study performed *in vivo* using melanoma cells is particularly informative on these stimuli (93). The authors have compared three different apoptotic signals and have confirmed that UVB-irradiation generates non-immunogenic apoptotic cells. Furthermore, the authors have identified that the production of IL-27 and IL-1α by bone marrow-derived macrophages after *in vitro* incubation with apoptotic tumor cells predicts immunogenicity. In addition, this work shows that primary necrotic cells induced by tuberculosis-necrotizing toxin *in vivo* are also non-immunogenic (93). This confirms that necrotic cells induced by a repeated freeze/thaw procedure or obtained by incubating apoptotic cells for 24 h before infusion are very poor inducers of CD8^+^ T cell responses *in vivo* (94). The immunogenicity of necrotic cells remains, however, a matter of debate (92) that we do not want to comment further here; (*iii*) one infusion appears sufficient whereas multiple infusions may expose to a risk of immunization against apoptotic cell-derived antigens (95), as discussed in Ref. (92). The question arises as to how long the therapeutic effect will last. In the CIA model, the therapeutic effect of early-stage apoptotic cell infusion is transient but prolonged when associated with anti-TNF therapy (20). Only clinical studies will allow to answer to this question; and (*iv*) a systemic administration route can be considered while local administration is also possible. In experimental arthritis models, two distinct systemic routes of administration [*i.e*., i.p. (13, 14) *versus* i.v. (13, 15, 20, 48)] have been tested with a similar efficacy (Table 1). However, no local administration has been evaluated so far. Another lesson coming from cancer research on dying/dead cells is the ability of apoptotic tumor cells to stimulate the proliferation of nearby viable tumor cells (96, 97). This apoptotic cell-mediated proliferation is not restricted to tumor cells (98). Relevant to the present review, primary human synovial fibroblasts isolated from knee joints of RA patients are also able to proliferate *in vitro* when these fibroblasts are in close contact with apoptotic tumor cells (97). Considering the therapeutic use of apoptotic cell infusion, it is, however, reassuring to see that when the number of apoptotic tumor cells is increased, the proliferative effect is limited (97). The percentage of infused early-stage apoptotic cells planned to be infused to RA patients is higher than 50%. However, one has to be cautious on this apoptosis-induced proliferative effect, since it is mainly mediated by a soluble factor, the nucleoside inosine (97).

Finally, one has to remain cautious, since a different effect can be obtained depending on the infusion of “resting” *versus* “activated” apoptotic CD11c^+^ cDC (48). This may be related to the methylation status of DNA from apoptotic cells (91). This work found that, as apoptotic cDC, apoptotic CD4^+^ T cells from RA patients exhibit a DNA demethylated status, suggesting a pro-inflammatory effect after infusion associated with IL-6 secretion (91). Whether this may impact on the therapeutic efficacy of apoptotic cell infusion remains to be determined. We used apoptotic splenic cells from arthritic mice (*i.e*., containing multiple activated leukocytes) in the therapeutic CIA model, and we observed the same effects as apoptotic thymocytes (Bonnefoy F., Perruche S., unpublished results). An additional security can be also provided by the addition of csDMARD, such as low-dose MTX or bDMARD, such as TNF inhibitors. Indeed, these treatments do not inhibit the beneficial therapeutic effects of apoptotic cell infusion (20). MTX (at high-dose) has been also used as prophylaxis of graft-*versus*-host disease in the clinical trial testing the effects of donor early-stage apoptotic cell infusion (16). This confirms our experimental data in CIA: MTX does not affect the therapeutic effect of apoptotic cell infusion, and allows to preserve its beneficial effect on collagen (autoantigen)-specific Treg (20). In addition, the capacity of splenic pDC and macrophages to induce *ex vivo* pTreg polarization is not inhibited by MTX (20). Thus, MTX can be continued if an apoptotic cell-based therapy has to be proposed to patients. Compared with MTX, anti-TNF therapy has the advantage to synergize with apoptotic cell infusion to control ongoing arthritis (20). However, the exact mechanism responsible for this synergy has not been identified (20). In the future, IL-6 inhibitors, such as tocilizumab, can be also envisaged to be associated with apoptotic cell infusion in order to prevent the antagonistic effect of IL-6, previously reported in mBSA-induced arthritis (48, 91). This can be a way to neutralize the effects of the methylation status of DNA from apoptotic T cells obtained from RA patients (91).

Apoptotic cell infusion can potentially be associated with corticosteroids without any risk. Indeed, corticosteroids enhance apoptotic cell removal by inducing the expression of the PtdSer-binding protein, milk fat globule-EGF factor 8 (MFG-E8) selectively in human and mouse monocytes and macrophages (whatever their differentiation profile, M1 or M2) (99).

## Conclusion/Concluding Remarks

Apoptotic cell infusion represents an additional potential bDMARD in RA, and more particularly a cell-based bDMARD. We plan to initiate a phase I/II clinical trial (ClinicalTrials.gov Identifier: NCT02903212) to achieve LDA in patients with RA who did not respond adequately to one previous bDMARD. Concerning the potential toxicity of this approach, one may build on the experience gained by the clinical trial performed in the setting of hematopoietic cell transplantation (16), but also those using extracorporeal photopheresis (ECP) in RA patients (100, 101). Even if ECP does not necessarily generate “proper/pure” early-stage apoptotic cells (102), this treatment introduces high amounts of dead cells in patients and no specific toxicity has been reported (100, 101). The efferocytosis capacity of monocyte-derived macrophages from 14 RA patients has been shown to be similar to those of healthy volunteers (103). A careful selection of patients should be done in order to avoid genetic alterations of molecules involved in efferocytosis (e.g., MFG-E8), as well as apoptotic cells carrying demethylated DNA (91). One advantage of cell-based therapies could be the adherence to treatment since we propose only one infusion in our clinical trial. This study is an opportunity to analyze in human the immune mechanisms triggered by infused apoptotic cells. Furthermore, association with biologic agents acting on different therapeutic targets (e.g., TNF) appears feasible to increase efficacy without the toxicity.

## Author Contributions

PS, FB, ET, and SP analyzed and discussed the literature and conceived the outline of the manuscript; PS wrote the manuscript. All authors edited the manuscript and provided valuable discussions and criticisms.

## Conflict of Interest Statement

The authors declare that the research was conducted in the absence of any commercial or financial relationships that could be construed as a potential conflict of interest.
